# Quantitatively Analyzing Pressure Induced Phase Transformation by Photoluminescence Spectra in Eu^3+^-doped Sodium Potassium Bismuth Titanate

**DOI:** 10.3390/ma13040881

**Published:** 2020-02-16

**Authors:** Liang Zeng, Ji Zhou

**Affiliations:** State Key Laboratory of New Ceramics and Fine Processing, School of Materials Science and Engineering, Tsinghua University, Beijing 100084, China; zeng-l15@mails.tsinghua.edu.cn

**Keywords:** pressure, phase transformation, piezoelectric material, photoluminescence, rare-earth ions, quantitative analyses

## Abstract

(Na_0.8_,K_0.2_)_0.5_Bi_0.497_Eu_0.003_TiO_3_ (NKBET20) piezoelectric ceramic powders were prepared by the solid-reaction method. The phase structures of the NKBET20 powders under various pressures were investigated by photoluminescence (PL) spectra and X-ray diffraction (XRD). The PL spectra of the doped Eu^3+^ ions suggest a pressure induced transformation from the tetragonal to rhombohedral phase (R phase), and the phase transformations were confirmed by XRD analyses. Furthermore, the fluorescence intensity ratio of the D05→F27 transition to the D05→F17 transition (FIR_21_) could be utilized for the quantitative analyses of the phase transformation. The results from the PL method show that as the pressure increases from 0 to 500 MPa, the fractions of the R phase of the NKBET20 powders increase from about 11% to 58%, while the fractions of the tetragonal phase (T phase) decrease from about 89% to 42%, which are consistent with the XRD Rietveld refinement. Unlike the ceramic bulks, the pressure induced phase transformation in the ceramic powders shows no obvious trigger point and is much gentler. This work suggests a different viewpoint to study the pressure induced phase transformation qualitatively and quantitatively, which can be used for more phase analyses.

## 1. Introduction 

Piezoelectric material has been widely applied in numerous electromechanical devices. In fact, piezoelectric material usually works under mechanical pressures [1], thus, some researchers concentrate on the effects of mechanical pressures on piezoelectric material [2,3,4,5,6,7]. For example, Yao et al. reported that the piezoelectric coefficient decreases with increasing the mechanical pressures in the PbTiO_3_-based piezoelectric ceramic, which was further explained by the pressure-induced depolarization [4]. Pressure induced phase transformations are also reported widely [5,6,7,8,9]. Hall et al. suggested a phase transformation from the rhombohedral to the orthorhombic phase within the PbZrO_3_-PbTiO_3_ piezoelectric ceramic, induced by pressure [3]. Dong et al. found that pressure could drive (Na_1/2_Bi_1/2_)TiO_3_-based ceramics from the ferroelectric to the relaxor phase [7]. Pressure induced phase transformations in piezoelectric ceramic bulks have been studied extensively, however, the effects of pressures on piezoelectric ceramic powders are seldom considered, which are also of important scientific significance and practical applications. For instance, when grinding, the influence of the mechanical pressure upon piezoelectric ceramic powders is vital in the X-ray diffraction analyses, as pressure induced phase transformation often occurs in the piezoelectric material. Moreover, the properties of the ceramic bulks and powders with the same compositions may differ, so, in this present work, we focus on the effects of pressures on piezoelectric ceramic powders, which are seldom considered.

As a Pb-free piezoelectric material, the (Na_1−x_,K_x_)_0.5_Bi_0.5_TiO_3_ (NKBT100x) ceramic has been extensively investigated for its superior electrical properties [10,11,12]. The NKBT100x ceramic crystallizes the R phasein the Na_0.5_Bi_0.5_TiO_3_-rich compositions, and crystallizes the T phase in the K_0.5_Bi_0.5_TiO_3_-rich compositions [13]. While in compositions with x located at 0.16–0.2, the NKBT100x ceramic forms a morphotropic phase boundary (MPB) [14,15]. In these critical compositions, the R and T phases coexist, and NKBT100x ceramic exhibits optimal piezoelectric properties [13,15]. Furthermore, the Gibbs free energy gap between the two phases is small [16], therefore, phase transformation often occurs. For example, it is reported that phase transformation induced by electric fields occurs within the NKBT20 ceramic [17]. In addition, as described above, mechanical pressures could also induce phase transformations in piezoelectric materials. Thus, it seems that pressures could induce a phase transformation within NKBT100x materials. 

On the other hand, piezoelectric materials doped with rare-earth (RE) ions have received significant consideration [18,19,20,21,22]. The crystallographic symmetry of the host material is one of the most important factors affecting the photoluminescence (PL) property of RE ions. Even if RE ions are doped in a dilute concentration, enough PL signals can be obtained because of their efficient emission. In such concentrations, RE ions hardly influence the initial structures of the host material, while their PL signals could reflect the structural information of the host material; thus, RE ions can be used as probes [23,24]. Pr^3+^ ions were used to detect the phase transformation of (Ba_0.77_Ca_0.23_)TiO_3_ materials [25]. Er^3+^ ions were used to probe the phase structures in Pb-based piezoelectric materials [26]. Furthermore, the PL spectra of Eu^3+^ ions were utilized for quantitative analyses of the phase structures of the (Na,K)_0.5_Bi_0.5_TiO_3_:Eu piezoelectric materials in our earlier work [27]. Here, we try to use the PL method for phase analyses in the pressure induced phase transformation.

In this contribution, we fabricated (Na_0.8_,K_0.2_)_0.5_Bi_0.497_Eu_0.003_TiO_3_ (NKBET20) piezoelectric ceramic powders by a solid reaction method, and investigated their phase structures under various pressures by PL spectra and XRD. The PL spectra of doped Eu^3+^ ions suggest that pressures induced the increase of the fraction of the R phase, and the decrease of the fraction of the T phase. Unlike the ceramic bulks, the pressure induced phase transformation in ceramic powders shows no obvious trigger point and is much gentler. Furthermore, FIR_21_ were shown to quantitatively analyze the phase transformation. These analyses were further confirmed by the XRD results.

## 2. Materials and Methods 

(Na_1−x_,K_x_)_0.5_Bi_0.497_Eu_0.003_TiO_3_ (NKBET100x; x = 0.1, 0.2, and 0.3) ceramic pellets were fabricated by the solid-reaction method, as described elsewhere [27]. Next, ceramic pellets were ground to a powder and annealed at 600 °C for 2 h. Then, the ceramic powder of NKBET20 was pressed into a stainless-steel die for 30 min under various pressures, ranging from 0 to 500 MPa, which was loaded by a tablet machine (DY-30, Keqi Ltd., Tianjin, China). The XRD measurements were executed using the Rigaku D/max-2500H X-ray diffractometer, which works under 40 kV and 150 mA. The scan angle ranged from 20° to 120°, with an interval of 0.01°. A spectrophotometer (FLSP920, Edinburgh Instruments, Livingston, UK) was used to record the PL properties. The excitation wavelength was set at 525 nm. For the PL spectra, the monitored luminescence range was from 570 to 645 nm with a bandwidth of 0.2 nm, and for the decay curves, the monitored wavelength was 592 nm.

## 3. Results and Discussions

**Figure 1:**
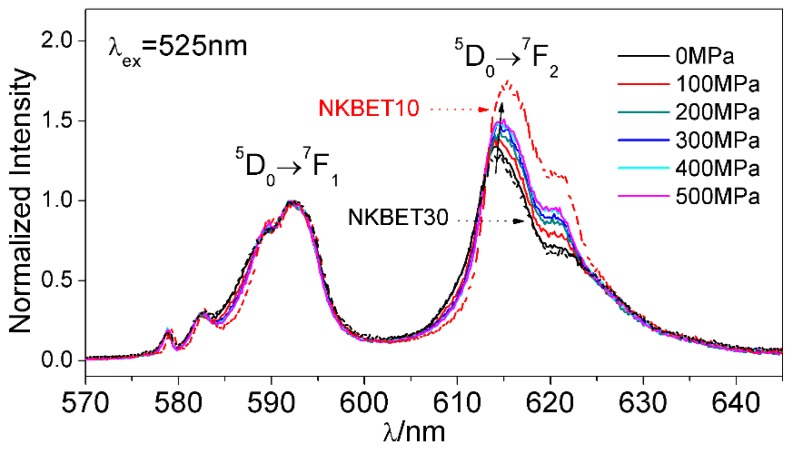
Photoluminescence (PL) spectra of the (Na_0.8_,K_0.2_)_0.5_Bi_0.497_Eu_0.003_TiO_3_ (NKBET20) ceramic powders under various pressures. The peak intensity of the magnetic dipole (MD) transition D05→F17 is normalized to 1; the dashed lines represent the PL spectra of the NKBET10 and NKBET30 ceramic powders; the black arrow represents the variations of the PL spectra.

Figure 1 depicts the PL spectra of the NKBET20 ceramic powders excited at 525 nm under various pressures. The magnetic dipole transition (MD) D05→F17(585–600 nm) is independent of the local environments [28], while the so-called “hypersensitive transition” D05→F27(600–630 nm) is sensitive to the local environments [29]. Figure 1 shows that as the pressures increase, the fluorescence intensity of the D05→F27 transition (I_2_) increases. As Eu^3+^ ions present the same PL spectra when distributed in NKBET100x materials with the same phase [27], utilizing the NKBET10 and NKBET30 ceramic powders as the reference of the R and T phases, the variations of I_2_ suggest a transformation from the T to R phase.

**Figure 2:**
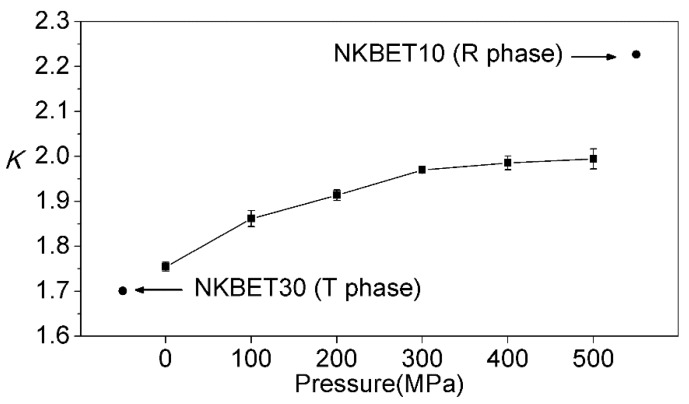
The fluorescence intensity ratios of the D05→F27 transition to the D05→F17 transition of the NKBET20 ceramic powders under various pressures, denoted as *K*. The two black dots represent the NKBET10 and NKBET30 ceramic powders as references.

Considering the sensitivity of the hypersensitive transition of D05→F27, and the independence of the MD transition of D05→F17, FIR_21_ is a good measure of the Eu^3+^ ions’ local environments. As the discussed intensity of the transition is the integral intensity, Lorentz profiles were used to fit the spectra so as to obtain accurate values [24,30]. As the pressures increases, the FIR_21_ of the NKBET20 ceramic powders increase from about 1.75 to 1.99, as shown in Figure 2. Using NKBET10 and NKBET30 ceramic powders as references, the increase of FIR_21_ also indicates a transformation from the T to R phase. In an earlier study [27], we utilized FIR_21_ to quantitatively analyze the phase structures of the NKBET100x with compositions at the MPB by Equations (1) and (2):

(1) $$K^{M}=\frac{\tau^{R}\alpha^{R}}{\tau^{R}\alpha^{R}+\tau^{T}\alpha^{T}}K^{R}+\frac{\tau^{T}\alpha^{T}}{\tau^{R}\alpha^{R}+\tau^{T}\alpha^{T}}K^{T}$$

(2) $$\alpha^{R}+\alpha^{T}=1$$

**Figure 3:**
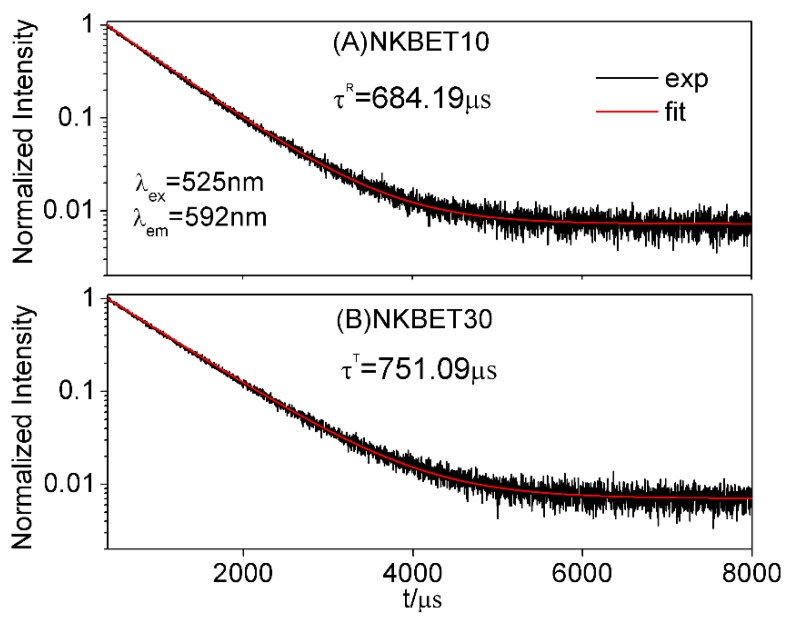
The decay curve of the ceramic powders of (**A**) NKBET10 and (**B**) NKBET30. The excitation wavelength is 525 nm and the monitored luminescence wavelength is 592 nm. The monoexponential function, I(t)=I(0)exp(−t/τ), was used to fit the decay curve in order to obtain the decay time.

Here, *K* is FIR_21_; *α* is the volume phase fraction; *τ* is the decay time; and superscripts M, R, and T represent the MPB, R, and T phase, respectively. $K^{R},\;K^{T},\;\tau^{R}$, and $\tau^{T}$ are calculated from the PL properties of the NKBET10 and NKBET30 compositions. $K^{M}$ is calculated from the PL spectra of the NKBET100x with compositions near the MPB, then the phase fractions of $\alpha^{R}$ and $\alpha^{T}$ can be quantitatively calculated via Equations (1) and (2). Similarly, this PL method could be applied in pressure induced phase transformations. The FIR_21_ of the NKBET20 ceramic powders under various pressures are shown in Figure 2. The decay time of the R and T phases (using NKBET10 and NKBET30 compositions as references) are used to correct the phase fractions according to the analyses of the previous work [27], obtained from the decay curves of the NKBET10 and NKBET30 ceramic powders, as shown in Figure 3. Using NKBET10 and NKBET30 ceramic powders as references, $K^{R},\;K^{T}$, (2.227 and 1.701, Figure 2), $\tau^{R},\;\mathrm{and}\;\tau^{T}$ (684.19 μs, 751.09 μs, Figure 3) in the above equations are identified. Then, the phase fractions of the NKBET20 powders under various pressures can be calculated by inputting *K*, thus solving Equations (1) and (2). 

**Figure 4:**
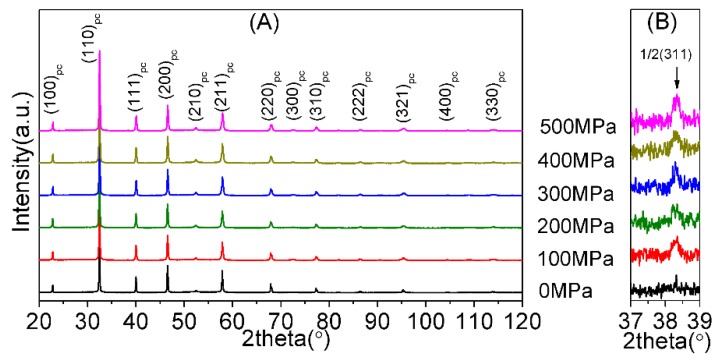
XRD patterns of NKBET20 ceramic powders under various pressures. (**A**) Ranges from 20° to 120°. (**B**) The superlattice reflection 1/2(311) corresponding to the space group R3c.

**Figure 5:**
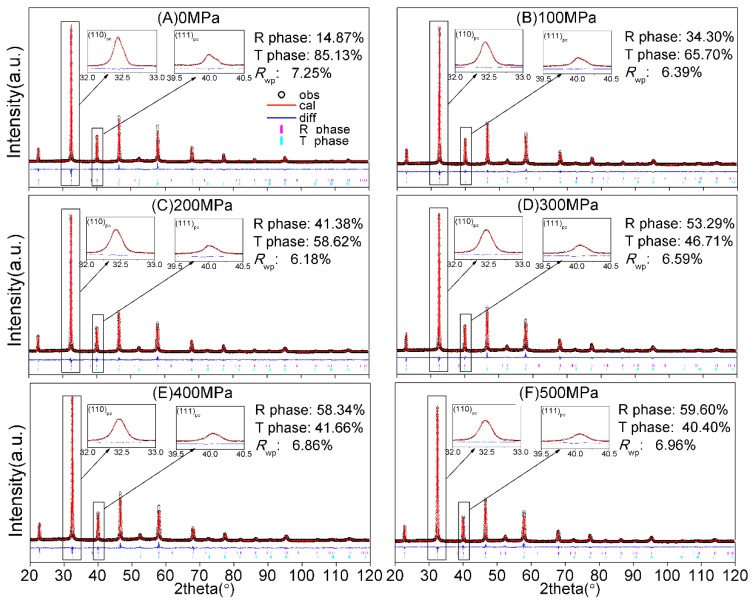
XRD Rietveld refinements of the NKBET20 ceramic powders under various pressures: (**A**) 0, (**B**) 100, (**C**) 200, (**D**) 300, (**E**) 400, and (**F**) 500 MPa.

XRD patterns are also utilized to analyze the phase structures of the NKBET20 ceramic powders under various pressures, as shown in Figure 4. Variations in the XRD patterns in Figure 4A suggest a phase transformation. Figure 4B shows the super-lattice reflection 1/2(311), which is related to the a^−^a^−^a^−^ tilting system of the space group R3c of TiO_6_ octahedral, with respect to other adjacent unit cells, giving rise to the super-lattice reflection [31,32,33]. The super-lattice reflection 1/2(311) could be used to confirm the R phase, as has been widely reported [34,35]. As the pressures increase, the intensity of the 1/2(311) reflection increases, suggesting that pressures induce the increase of the fraction of R phase (R3c). In addition, XRD Rietveld refinement was executed by the general structure analysis system (GSAS) for quantitative phase analyses [36,37], as shown in Figure 5, in which R3c (R phase) and P4mm (T phase) were utilized in the meanwhile [33,38]. The fitted parameters are summarized in Table 1. From Figure 5 and Table 1, it can be seen that all of the patterns are fitted well.

**Figure 6:**
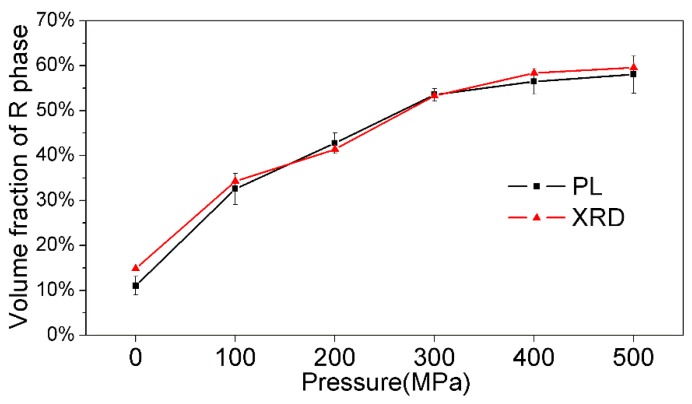
Variations of the fraction of the R phase of the NKBET20 ceramic powders under various pressures.

Figure 6 depicts the variations of the phase fraction of the NKBET20 ceramic powders as the pressures increase. It can be seen from the results of the PL method that as the pressure increases from 0 to 500 MPa, the fractions of R phase of the NKBET20 powders increase from about 11% to 58%, while the fractions of the T phase decrease from about 89% to 42%. The phase analyses from PL method were consistent with the XRD Rietveld refinements. The phase transformation induced by the pressures within the piezoelectric ceramic bulks usually presents a trigger point and sharp variation [39,40]; however, the piezoelectric ceramic powders show no obvious trigger point and the phase transformation is much gentler. This finding indicates that the grind of the piezoelectric ceramic powders may induce a phase transformation, which needs additional care when doing the XRD measurements. In addition, the pressure induced phase transformation could be detected by the PL method, indicating the potential for Eu^3+^ ions to be used as in site probes for phase transformations. The experiments set up for PL detection are easy to build, which can be home-made to satisfy various demands, like electric field module, pressure module, and temperature module. Compared with the XRD Rietveld refinements, which need demanding devices and precise patterns, the PL method is a simple and fast procedure. We show that the PL method could be applied in pressure induced phase transformation in this work, yet it has much potential in fields of other phase analyses.

## 4. Conclusions

In summary, pressures induce a phase transformation within the NKBET20 ceramic powders, and the PL properties of Eu^3+^ ions can be utilized to analyze the transformation qualitatively and quantitatively. Utilizing NKBET10 and NKBET30 ceramic powders as references, the increase of I_2_ suggests a pressure induced transformation from the T to R phase. Furthermore, FIR_21_ were shown to quantitatively analyze the phase transformation. The results from the PL method show that as the pressure increases from 0 to 500 MPa, the fractions of the R phase of NKBET20 powders increase from about 11% to 58%, while the fractions of the T phase decrease from about 89% to 42%. Both the qualitative and quantitative phase analyses were further confirmed by the XRD results. Unlike the ceramic bulks, the pressure induced phase transformation in the ceramic powders shows no obvious trigger point and is much gentler. This work suggests a different viewpoint to study the pressure induced phase transformation, both qualitatively and quantitatively, which can be used for more phase analyses.

## Figures and Tables

**Table 1:** The parameter of XRD Rietveld refinement.

*P* (MPa)	R3c				P4mm				*R* Factors	
	*a* (Å)	*c* (Å)	*V* (Å^3^)	*vol*%	*a* (Å)	*c* (Å)	*V*(Å^3^)	*vol*%	*R_wp_*%	*R_p_*%
0	5.5170	13.5200	356.377	14.87	3.9032	3.9111	59.586	85.13	7.25	5.73
100	5.5167	13.5171	356.263	34.30	3.9031	3.9100	59.566	65.70	6.39	4.95
200	5.5162	13.5137	356.116	41.38	3.9029	3.9096	59.555	58.62	6.18	4.79
300	5.5150	13.5133	355.946	53.29	3.9029	3.9092	59.548	46.71	6.59	5.06
400	5.5145	13.5135	355.892	58.34	3.9023	3.9079	59.514	41.66	6.86	5.28
500	5.5139	13.5129	355.798	59.60	3.9021	3.9071	59.490	40.40	6.96	5.26
